# Development and accuracy of a hearing screening application

**DOI:** 10.1016/j.bjorl.2020.03.009

**Published:** 2020-05-05

**Authors:** Magda Aline Bauer, Afonso Sales, Adriane Ribeiro Teixeira, Patrícia Morsch, Alexandre Hundertmarck Lessa, Ângelo José Gonçalves Bós

**Affiliations:** a Pontifícia Universidade Católica do Rio Grande do Sul (PUCRS), Gerontologia Biomédica, Porto Alegre, RS, Brazil; b Pontifícia Universidade Católica do Rio Grande do Sul (PUCRS), Porto Alegre, RS, Brazil; c Universidade Federal do Rio Grande do Sul (UFRGS), Porto Alegre, RS, Brazil; d Faculdade de Educação e Meio Ambiente (FAEMA), Ariquemes, RO, Brazil; e Pontifícia Universidade Católica do Rio Grande do Sul (PUCRS), Escola de Medicina, Porto Alegre, RS, Brazil

**Keywords:** Audiometry, Hearing loss, Screening programs, Mobile applications

## Abstract

**Introduction:**

Hearing impairment, or hearing loss, can be caused by several factors and the implications vary according to the type, degree, cause and age of occurrence. Hearing screening should be a common procedure, allowing the pre-clinical identification and necessary referrals, avoiding the consequences of auditory deprivation. Mobile apps have shown to be a good alternative for hearing assessment.

**Objective:**

The objective was to develop an app and assess its performance in identifying hearing loss by comparing it with another validated screening tool.

**Methods:**

The application, called *Ouviu*, was created using audiological knowledge and tools available on the iOS platform. We evaluated 185 people, aged between 6 and 96 years, distributed into 5 age groups, performing audiometry and hearing screening using two tools: HearCheck and the application.

**Results:**

The results showed that the sensitivity of the application in identifying hearing loss was approximately 97%, while that of HearCheck was 79%. The positive predictive value of the application showed that the probability of a person being identified with this tool and actually having a hearing loss was 94%, while for HearCheck it was 96%. False negatives, which failed hearing loss identification, were fewer in the app (3%) than in HearCheck (21%).

**Conclusion:**

Consequently, the developed application was shown to be effective as a hearing screening tool, surpassing HearCheck in identifying mild hearing loss. In addition to being portable, easy to apply, low cost and rapidly performed, the application has the advantage of assessing environmental noise to perform the exam, as well as the fact that it is not necessary to attach any hardware to the mobile device.

## Introduction

Hearing is an extremely complex function, being essential for the processing of acoustic events and for the emission and understanding of speech signals. Hearing loss can be caused by several factors and, among them, the prenatal etiologies such as genetic inheritance, inner ear malformations, congenital infections by the rubella virus, cytomegalovirus, herpes, toxoplasmosis and syphilis; perinatal causes, such as anoxia, prematurity, hyperbilirubinemia, head trauma and sound trauma; and postnatal problems, including metabolic causes such as hypothyroidism and diabetes, viral infections such as rubella, chickenpox, influenza, mumps, cytomegalovirus, bacterial meningitis, encephalitis and chronic otitis media.^1^ Hearing loss can also be caused by excessive exposure to noise^2^ and multiple factors that have an impact on the inner ear throughout life, which cumulatively lead to presbycusis.^3^

The consequences of hearing loss vary according to the type, degree, cause and age of its occurrence. Linguistic, educational and psychosocial impairments can be observed in children and adolescents.^4^ In adults and the elderly, social isolation is usually observed, with restrictions regarding one's participation in social and family life, often as an attempt to avoid ridicule or contempt.^5^ Hearing loss can also be associated with cognitive decline, depression and reduced functional status.^6^

Hearing loss diagnosis and rehabilitation should be attained early, both in children and in individuals at other stages of life. However, hearing loss occurs gradually, with a slow progression in many situations and it is often not perceived or denied by the individual.^5^ Thus, hearing screening should be a common procedure in schools, hospitals, health clinics, clinics and even at home, aiming at testing a large number of individuals quickly, simply and cost-effectively, allowing an early diagnosis and the necessary referrals, avoiding the consequences of hearing deprivation.

Currently, screening can be performed using standardized questionnaires^7^ or by performing simplified hearing tests, which do not aim at determining hearing thresholds, but rather at identifying the possibility that the individual may have hearing loss. They can also be performed by screening tools, such as the HearCheck Screener hearing screening equipment, which can be used on individuals in the most diverse age groups. It was designed to be used in quiet environments, without requiring the use of an acoustic booth. The device operation is simple and does not bring any discomfort to the patient. The examiner must take note of the sounds perceived by the individual, and the perception of fewer than three sounds indicates that the patient should be referred for a complete audiological evaluation.^8^ However, despite the benefits already verified for this screening equipment, the device has relatively high costs, has limitations in the number of performed tests and evaluated frequencies (1000 and 3000 Hz), in addition to requiring qualified professionals for its use.

Hearing screening is an already established concern, and several alternatives have been suggested for this purpose. This concern is no different with the elderly population. We know that access to hearing tests is not a reality for this population. Displacement, referrals, qualified professionals and places for exams are some of the barriers faced by this age group. Therefore, in the Elderly Booklet (*Cartilha Idosa*), the use of the Whispered Voice Test was proposed as an integral part of the measurements that should be performed in the elderly. However, Bauer et al. (2017)^9^ assessed long-lived elderly with this test and compared the results with those of the Hearcheck and did not obtain satisfactory results, suggesting that the Whispered Voice Test, as indicated to be performed, fails to accurately detect possible hearing losses.

Therefore, knowing the need for an instrument that would meet this demand, we found in the literature that applications for mobile devices have shown to be a good alternative for performing auditory screening, since they have the benefit of being less expensive, showing better acceptance among users and health professionals, simplified application and registration, in addition to being portable and not requiring periodic assistance. The applications (apps) are available for download on iOS and Android platforms, and many of them are free of charge. Some have already been tested with positive results, while others.lack careful study.^10^, ^11^, ^12^

Despite the existence of some applications, we saw that some issues made them difficult to use on a larger scale. The main one was the use of headphones, since the need to have hardware like this makes it difficult to use, due to the impositions of cleaning after use, of having good-quality headphones, of having a more invasive contact with the assessed individual and greater expense. We also did not find national apps validated for the Brazilian population that were free of charge. Moreover, most existing applications seek to replicate audiometry, screening all frequencies. As our intention was to screen, we wanted something more concise that could be used by other professionals who do not necessarily have audiological knowledge and that would take into account the environmental noise at the testing site.

Observing the need for a differentiated screening instrument that would assist in the screening of hearing acuity, we considered developing an application, believing that this would prove to be a simple, inexpensive, effective, safe and quick way to identify possible losses. Therefore, the present study main objective was to develop and evaluate the performance of a hearing screening application in different age groups (for that, the specific objectives were: to develop a hearing screening application, to establish normality standards for the application, to verify the application performance in identifying HL in different age groups and checking the performance of HearCheck in identifying HL in different age groups).

## Methods

The study, approved by the Scientific Committee of *Instituto de Geriatria e Gerontologia da Pontifícia Universidade Católica do Rio Grande do Sul* (PUCRS) and the Research Ethics Committee of PUCRS, CAAE 57742016.8.0000.5336, was a cross-sectional, observational, descriptive, and comparative one. The sample consisted of individuals aged from 6 years old onward who underwent hearing assessment, by their own initiative or by referral from health professionals, with the collection period comprising one year (August 2016 to August 2017).

The entire collection was carried out at Universidade Federal do Rio Grande do Sul (UFRGS), using the equipment of the Audiology Clinic of that institution.

The individuals were divided into groups with at least 38 research subjects in each – as per the sample calculation, according to Hajian-Tilaki (2014)^13^ – except the long-lived elderly group, which had 27 individuals (although the collection period was extended for one year, no more people in that age group were added). The following division was used: children and adolescents (6 to 19 years), young adults (20 to 39 years), middle-aged adults (40 to 59 years), young elderly (60 to 79 years) and long-lived elderly (age ≥ 80 years).

Subjects who had obstructive cerumen in the external auditory canal, uni- or bilaterally, were excluded. The individuals, after signing the Free Consent form, answered a personal data sheet with relevant information for the research and, subsequently, they were submitted to the evaluations. Meatoscopy exam, hearing screening with the Hearchek Screener equipment (Siemens), auditory screening with the developed application and pure tone audiometry were performed.

Meatoscopy aimed to verify the absence of obstructive cerumen in the participants’ auditory canal, and it was the first examination performed. Hearing screening with the HearCheck equipment was performed according to the manufacturer's standards and for the analysis of the results, the criteria proposed by Cardoso et al. (2014) was employed.^8^ This instrument screens the frequencies of 1000 and 3000 Hz at three intensities.

The application, called “*Ouviu*”, was developed on the iOS platform to perform auditory screening. This evaluation instrument was developed in partnership with *Escola Politécnica* of PUCRS. It consists of the following procedures: Environmental noise analyzer (measurement of environmental noise), test (screens the frequencies of 1000, 2000, 4000, 8000 and 500 Hz, at the “Moderate”, “Weak” and/or “Strong” intensities) and results (tab with individual responses to the presented sounds, and according to the intensity heard or not). We chose to present the sounds in a free field, and not through headphones. To evaluate the results of the application, tests were considered negative when the participants heard the frequencies of 1000, 2000 and 4000 Hz at 20 dB and at the frequencies of 500 and 8000 Hz at 40 dB, whereas they were considered positive when the participants did not hear in at least one of the mentioned frequencies and intensities. This criterion was built based on the hearing thresholds within the normal range obtained at the audiometry of the first 10 participants of each age group.

The audiometry was performed and analyzed according to the World Health Organization (WHO) standards.^14^ For the purposes of this research, we chose to use the degrees of hearing loss (mild, moderate and severe) in the analyses, and not the type. Also, because the test with the application was performed in a free field (both ears simultaneously evaluated), we chose to use the audiometry result of the best ear.

After performing the tests, the data were entered into a database created in Microsoft Excel. The analyses were performed using the EPIINFO software, with the aim of analyzing the association between the assessed variables.

## Results

### Application development

The application was developed on the Xcode development platform, version 9.4.1, using the Swift programming language, version 3.3. The AVFoundation's native video and audio manipulation framework (https://developer.apple.com/av-foundation/) was used. The used concept was the amplitude (Float value that varies from 0 to 1). Frequency and intensity tests of the waves generated by the speaker built into the fourth generation iPod Touch device were performed. Two test batteries were performed, one to understand the physical limitations of the device in generating the frequencies (500, 1000, 2000, 4000 and 8000 Hz) and the second for the desired intensities (20–40–60 dB). The amplitude was controlled through an application that allowed changing it according to the power measured in the decibel meter. The decibel meter used was provided by LABELO (Specialized Electro-Electronics Laboratory at PUCRS) in a test room with sound insulation. Thus, it was possible to adjust the application to work on the iOS platform with the following adjustments: at the maximum output intensity of the equipment (iPod Touch), at the frequencies of 1000, 2000, 4000 and 8000 producing pure tones at the intensities 40 dBHL and 60 dBHL, and the frequency of 500 Hz producing 40 dBHL (60 dB production was not supported by the equipment). These intensity values were measured for the moment they reached the subject and not when they left the equipment. Intensities below 40 dBHL could not be measured, due to the wave characteristics and their dispersion in the environment. But they were programmed to be produced from visual and auditory adjustments of the waves produced by the equipment and the perceptions of the involved professionals.

Aiming to facilitate the test understanding, not to confuse users, nor to erroneously infer values for the auditory findings, we chose to name the intensities as “Weak” (lower than 40 dBHL), “Moderate” (40 dBHL) and “Strong” (60 dBHL). It should be noted that it was decided not to use headphones and simultaneous testing between the ears, since the use of headphones would imply costs and the need for sanitation between the participants’ use (which would make it difficult to use in the primary or home care). The absence of headphones made it difficult for the intensity generated by the source (in this case, the iPod) to be the same as the one that reached the subject, causing the need for adjustments so that the intensity generated was sufficient to reach, for instance, 40 dB in the ears. This problem meant that we could not state that intensities below 40 dB would be measured, since the environmental noise is mistaken by the produced sound. For this purpose, we chose to adjust the output with algorithms that were visually and audibly less than 40 dBHL. HearCheck, for example, emits a 1000 Hz pure tone at 35 dBHL as a minimum value.

After some versions and measurements in a specific laboratory, and also due to the precise specifications of the iPod hardware, the frequencies and intensities were distributed as follows: the frequencies of 1000, 2000 and 4000 Hz present the moderate and strong intensities confirmed at 40 and 60 dB, respectively, and weak (lower than 40 dB, but with no possibility of stating the intensity); the frequency of 8000 Hz with all confirmed intensities; and the frequency of 500 Hz with the assessed moderate intensity, the weak intensity without confirmation, and the strong intensity (60 dB) was not supported by the equipment (not being produced).

As for the application results, tests were considered negative when the participants heard the frequencies of 1000, 2000 and 4000 Hz at moderate and weak intensities, and the frequencies of 500 and 8000 Hz at moderate intensity. And they were considered positive when the participants did not hear at least one of the frequencies and the intensity was at least moderate. This criterion was built from the hearing thresholds within the normal range obtained in audiometry. Participants with normal hearing were the beacons of negative results in the application, and those with HL of different degrees were the positive result for the same device.

The test application time – relevant data, since the demand in primary and home care is high – was measured in some applications, with an average time of 30 s, which may be shorter if the patient responds quickly, and up to 45 s, if the patient does not answer to any intensity (maximum time). For comparison purposes, the time used in HearCheck was also measured. The test is performed within a pre-defined time by the device, with 40 s for each ear. Still thinking about primary care, the application shows advantages over the free tests available on the internet since it was validated, checks environmental noise and does not need a computer to be applied.

### Sample distribution

**Figure 1:**
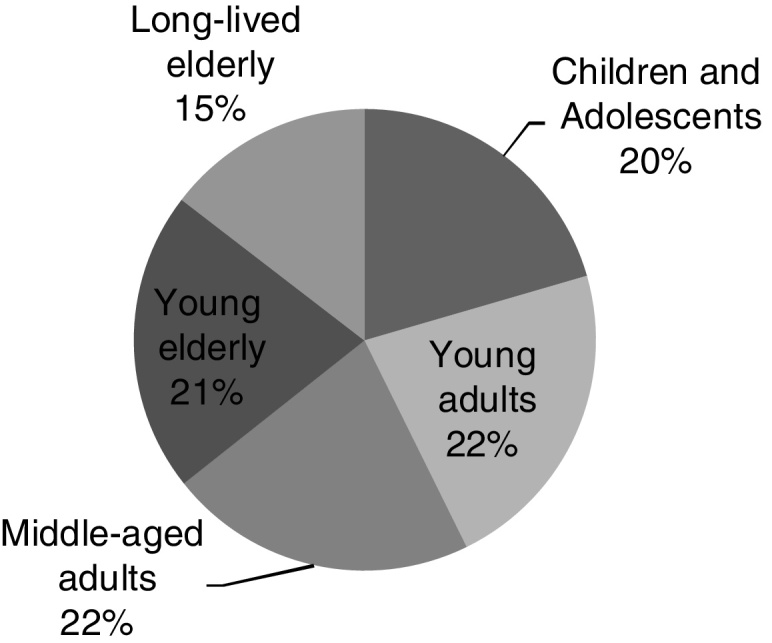
Sample distribution in the group by age range.

The sample (Fig. 1) consisted of 185 participants distributed in: 38 children and adolescents (between 6 and 19 years), 41 young adults (between 20 and 39 years), 40 middle-aged adults (between 40 and 59 years), 39 young elderly (between 60 and 79 years) and 27 long-lived elderly (aged 80 years or over). As for the participants’ gender, there were more females, 112 women (60.5%) and 73 men (39.5%): of the children and adolescents, 21 (55%) were females; of young adults, 28 (68%); of the middle-aged adults, 24 (60%); among the elderly, 23 (59%); and among the long-lived elderly, 16 were females (59%).

### Application performance

The results achieved are related to the sensitivity and specificity of the *Ouviu* and HearCheck applications. The positive predictive value and the negative predictive value are related to Hearing Loss. For the analyses, the result of audiometry (presence or absence of HL and HL levels – mild, moderate and severe) was used as a fixed variable. The following were considered: True Negative, for those who had a negative result for Hearing Loss (normal hearing) both in the screening (at the application or HearCheck) and in the audiometry; True Positive, for those who had Hearing Loss detected in both screening and audiometry; False Positive, for those who had a positive screening and normal hearing; and False Negative, for those who had a negative screening and, however, had hearing loss. The degree of loss was used for analysis of responses to screening (positive or negative) and presence or absence of HL for the other analyses.

Table 1 shows the total of 185 participants and the comparison of audiological findings (presence and absence of HL) with the results of the HearCheck and application screenings. Of the total number of assessed individuals, 117 (63%) audiological hearing results were found to be within the normal range, and 68 (37%) of HL; 40 (59%) of mild HL; 21 (31%) of moderate HL; and 7 (10%) of severe HL.

**Table 1:** Performance of the application and HearCheck in identifying hearing loss.

	*HearCheck*	APP
Sensitivity (%)	79.4	97.1
Specificity (%)	98.3	96.6
Positive predictive value (%)	96.4	94.3
Negative predictive value (%)	89.1	98.3
True positives (*n*)	54	66
True negatives (*n*)	115	113
False positives (*n*)	2	4
False negatives (*n*)	14	2
Accuracy (%)	91.4	96.8
**Total (*****n*****)**	185^a^	185^a^

a 117 individuals with normal hearing and 68 with HL (hearing loss). APP, application.

Both screenings were mostly sensitive to identify HL, with HearCheck sensitivity being 79% and the application's, 97%. Similarly, specificity also showed high values, 98% and 96% for HearCheck and app, respectively, with both instruments being able to assess participants with hearing levels within normal standards.

HearCheck's positive predictive value showed that the likelihood of a person being identified with this instrument and actually having HL was 96%. The application's rate was 94%. On the contrary, the negative predictive values of 89% and 98% showed the probability that the person who had a negative screening in fact did not have HL.

The true positives, screenings with positive results for HL and with HL, were 54 (79%) in HearCheck and 66 (97%) in the application. In contrast, the true negatives, negative screenings and absence of HL were 98% (115) with HearCheck, and 97% (113) with the app. The false positives, a positive screening for HL and absence of HL were 2 and 4 for HearCheck and app, respectively. The false negatives, which failed to identify HL, were 20.59% (14) for HearCheck, and 3% (2) for the app.

Finally, the accuracy of the screening in discriminating between HL and normal hearing in the entire sample was 91% for HearCheck and 97% for the application.

## Discussion

### Application development

When developing the application, the first concern we had was with environmental noise. Fearing that it would interfere with the test results, we placed a noise analyzer on the home screen. The analyzer aims at allowing the start of the test only when it detects ambient noise limits <60 dB. The concern with environmental noise at the place where the screening was applied was raised as being an important factor for the quality of the tests in other studies.^15^, ^16^ However, we did not find another application that made this tool available as a prerequisite for the screening.

The pure tones of the frequencies of 500, 1000, 2000 and 4000 Hz, and the intensities (weak, moderate and strong) were chosen considering those established by the WHO. The weak intensity, presented as lower than 40 dB, refers to the minimum intensity required within the normal range; moderate (at 40 dB) is an analogy to moderate hearing loss; and the strong one refers to deeper degrees of loss.^14^ The outstanding frequencies of our application, 500 and 8000 Hz, were chosen because they are warnings: the 500 Hz for conductive loss, more common in childhood,^17^ and the 8000 Hz in age-related HL.^3^ We chose three intensities of each frequency, and each subject performed only two, similar to the HearCheck, which emits a pure tone of 1000 Hz at 20, 35 and 55 dB, and 3000 Hz at 35, 55 and 75 dB. The other applications validated in the literature perform screenings at more frequencies (usually 250–8000 Hz) and at varying intensities.^10^, ^11^, ^12^, ^13^, ^14^, ^15^, ^16^, ^17^, ^18^, ^19^

The normality standards were established and considered the results of the audiometry. To have a negative test in the application, the subject should hear the moderate and weak intensities at the frequencies of 1000, 2000 and 4000 Hz, and at least the moderate intensity of 500 and 8000 Hz, thus suggesting normal hearing. Not hearing the moderate intensities was seen as indicating some degree of hearing impairment. The cutoff point of our study was similar to that proposed by Abu-Ghanem et al. (2016),^10^ who suggested the intensity of 40 dB at the frequencies of 250–6000 Hz.

The time of the test application, considered important for a screening tool, was 30 s, on average, and could be shorter if the patient responds quickly, and up to 45 s, if the patient did not respond to any intensity (maximum time). For comparison purposes, the time used in HearCheck was also measured. The test is performed in a pre-defined time by the device, being 40 s in each ear. The testing times for both tools are similar to those found in another validation study that measured time less than one minute (49 s) in children, and slightly longer than one minute (74 s) in adults.^18^ Mahomed-Asmail et al. (2016)^19^ also found that the test time with the application was faster than the conventional screening used with children.

### Participants

We evaluated 185 people aged 6–96 years within a one-year period. The findings were analyzed for the total number of participants and by age group. Only the long-lived elderly group did not reach the desired number of participants (38), despite the long collection time (12 months). As our objective was to evaluate patients who came to the Audiology Clinic, without interfering with the demand, we chose not to call any external participants.

In all age groups, women were the majority of the assessed individuals. Women were also the majority in the validation study of another application and comprised 71% of the sample.^18^

With the results obtained in audiometry, we found that most of the hearing results were within the normal range, followed by mild, moderate and severe HL. This percentage of HL observed in the study was higher than the global estimate of 5.3%;^20^ and the national estimate, of 5.1%.^21^ However, one must consider two issues: (1) the people treated at the clinic were either referred by health professionals or were undergoing speech therapy, and therefore may have had a greater degree of hearing impairment and (2) as it is a routine to treat speech therapy patients without hearing complaints, this number of HL could be even higher in school-age populations, for instance.

Regarding the age distribution, the group aged 6 to 19 years (children and adolescents) was the second age group with the smallest hearing loss, since the age group with the smallest loss was that of young adults. In the other age groups, the frequency of hearing loss increased with age, being present in all long-lived elderly individuals. Again, in our sample, among children, adolescents, adults and the elderly, we had a higher frequency of HL than that reported worldwide and nationally. As expected, there was an increase in the frequency of HL among the elderly and long-lived elderly individuals, which corroborates world and national data.^20^, ^21^ A factor that should be considered, in addition to the profile of those evaluated in our study, is that in epidemiological research, information is self-reported and not measured clinically. The same justification was given in a literature review article that showed, among the adolescents’ reports, a prevalence lower than 2% of HL, while, among those who had the HL clinically proven through the audiometry, the prevalence was between 11.5 and 15.8. Our prevalence was among what was found by these authors.^22^ Another study that found differences between self-reported and proven data was one performed with the elderly, which had a prevalence equal to ours for this age group.^23^

### Performance of screening instruments

In general, HearCheck and the app performed similarly in all age groups. The first one was better at identifying normal hearing, and the second at identifying HL. HearCheck failed to identify mild HL but was effective in the higher degrees of HL. The application, in turn, was sensitive in all degrees of HL. In the joint analysis, with all age groups, where the majority showed hearing degrees within the normal standards, the performance of the tests was satisfactory. Both screenings were mostly sensitive to identify HL, with HearCheck sensitivity being lower than that of the application. Likewise, specificity also showed high values for both tests, being able to identify participants with hearing within normal standards. HearCheck overall sensitivity was lower than the instrument validation test, and the specificity was similar.^8^ This better performance of the application can be justified by the greater number of frequencies and intensities assessed, when compared to HearCheck.

As for the application, in a study with participants aged between 3 and 97 years old, quite different age groups such as in our sample, the sensitivity found was lower (81.7%) than that of our application, as well as the specificity (83.1%).^18^ HearCheck positive predictive value showed that the likelihood of a person being identified by this instrument and actually having HL was high, as well as with the app. On the contrary, the negative predictive value showed that the probability of people who had a negative screening did not in fact have HL was also high. Both predictive values were higher than those found in a study with another application, which were: positive predictive value of 87.6%, and negative predictive value of 75.6.^18^ As for the false negatives, considering HL that would go unnoticed in the screening and constitute a matter of concern regarding the test efficiency, HearCheck showed a seven-fold higher number of false-negatives than the *Ouviu* application. The accuracy of the screenings showed that they were vigorous in discriminating between HL and normal hearing in the entire assessed sample.

Our limitations regarding the platform on which it was developed comprise the access restrictions of mobile devices other than the iPods where the tests were performed. These limitations are also our future suggestions for improving the application for wider use on other platforms. Another limitation was the fact that the 50 participants whose audiometry results were used to establish the *Ouviu* normal hearing criteria were included in the analyses. New evaluations of the application can confirm the obtained results.

The development, which comprised from determining the frequencies and intensities that would be presented, through the form of display and adjustments to the layout and recording of the results, was designed to cover the “flaws” observed in other available applications. When we chose not to use headphones, we had problems with the calibration of the intensities, the reason why we were unable to state at what intensity it was working with the “weak” intensity. However, we did not have to worry about headphones hygiene, standardization and the costs they would bring.

Regarding the establishment of normality standards, considering the results of audiometry, we proposed that: listening to moderate and weak intensities at the frequencies of 1000, 2000 and 4000 Hz, and at least the moderate intensity of 500 and 8000 Hz is suggestive of normal hearing. On the other hand, not hearing the moderate intensities already indicates some degree of hearing impairment, and the subject must be referred to audiometry exams.

The application performance was satisfactory in all assessed age groups, as well as the HearCheck. Although both instruments were effective, the application showed a better accuracy than HearCheck and was more precise to identify HL of all degrees (including the mild degree). In addition to the previously described benefits, the application also shows advantages in terms of cost, speed, access to testing and applicability. Moreover, there are no portable devices available on the market that can generate calibrated sounds at the proposed frequencies and intensities.

We believe that the success, both in the development of the *Ouviu* app and in the results obtained with it, is due to the competence of the team involved in its development. Specific knowledge of computer science and audiology was shared and joined with efforts to create a tool that would be both viable and meet the clinical needs. The authors would like to thank Henrique Valcanaia and Marcos Vinícius Kuquert for the development of the application, which allowed the collection of data to be analyzed for this research. The participants who volunteered to take the exams, the students of the Speech Therapy course at Universidade Federal do Rio Grande do Sul who helped with data collection. This work was supported by *Coordenação de Aperfeiçoamento de Pessoal Nível Superior* – Brasil (CAPES), funding code 001.

## Conclusions

We developed an application that met our needs, capable of performing a quick HL screening with an effective performance in different groups, which included children as well as the long-lived elderly.

The final layout, proposed with the completion of data collection, analysis and result rationale, will be completed and made available on Apple Store.

## Funding

Study supported by Coordenação de Aperfeiçoamento de Pessoal Nível Superior – Brasil (CAPES) – funding code 001.

## Conflicts of interest

The authors declare no conflicts of interest.
